# Three-year follow-up case report: root canal treatment combined with intentional replantation for treating type III palatogingival groove in a maxillary lateral incisor

**DOI:** 10.3389/froh.2024.1467327

**Published:** 2025-01-17

**Authors:** Jixu Jia, Miao Cheng, Sumeng Shi, Yanchun Qiao

**Affiliations:** Department of Stomatology, Aerospace Center Hospital, School of Aerospace Clinical Medicine, Peking University, Beijing, China

**Keywords:** dens invaginatus, periodontal-endodontic lesion, intentional replantation, root canal therapy, infection control

## Abstract

Palatogingival groove is a developmental anomaly that can lead to significant periodontal and periapical diseases, particularly in Type III, which extends to the apex and communicates with the periodontal membrane through the apical foramen. This case report details the management of a Type III palatogingival groove in a maxillary lateral incisor, combining root canal treatment and intentional replantation, followed by a 3-year follow-up to assess the mid-term efficacy of this approach. The patient presented with a narrow and deep periodontal pocket on the palatal side, destruction of the palatal root surface and labial bone plate, and a persistent sinus tract. Initial treatment involved controlling the intracanal infection using root canal treatment under a dental microscope. One week later, the tooth was extracted for *ex vivo* preparation, including apical retrofilling and segmented filling of the root surface groove, before replantation and stabilization with elastic fixation on the labial side. At the 2-month follow-up, the labial sinus tract had completely healed. The palatal area, which initially exhibited a probing depth of 10 mm, demonstrated an 8-mm gain in clinical attachment. Probing revealed no signs of bleeding or pus discharge. Radiographic examinations showed the disappearance of periapical radiolucency. Regular follow-ups at 12, 24, and 36 months indicated no discomfort, with radiographic evidence of new bone formation at the apex and gradual recovery of the periodontal membrane, achieving new periodontal attachment. This case demonstrates that root canal treatment combined with intentional replantation, with a focus on infection control, is an effective treatment method for managing Type III palatogingival groove in maxillary lateral incisors.

## 1 Introduction

Palatogingival groove (also known as the radicular-lingual groove or disto-lingual groove) is a developmental anomaly of the oral and maxillofacial region, with a reported prevalence ranging from 1.01% to 18% across different populations (1). This anomaly arises from the infolding of the inner enamel epithelium and Hertwig's epithelial root sheath during tooth development, leading to the formation of irregular depressions or grooves on the tooth surface (2). These grooves provide an ideal niche for bacterial accumulation, predisposing the tooth to caries and combined periodontal-endodontic lesions (3).

The palatogingival groove is categorized into three types based on its length, depth, and the complexity of the root canal system: Type I, a short groove confined to the coronal third of the root with normal root canal morphology; Type II, a long but shallow groove extending beyond the coronal third of the root, associated with a normal or relatively simple root canal configuration; and Type III, a long and deep groove penetrating beyond the coronal third of the root, characterized by a complex root canal system (4).

Managing palatogingival groove lesions requires a precise and personalized treatment approach based on the lesion's severity (5). Non-surgical interventions such as mechanical cleaning, medicated irrigation, and resin fillings may suffice for mild cases (6). For severe infections or cases with significant clinical symptoms like combined periodontal-endodontic lesions or periapical inflammation, multidisciplinary treatment is advocated. In cases where the lesion infects the pulp, root canal treatment is necessary. For teeth with significant periapical and periodontal destruction, apical and periodontal surgeries can promote healing of the periapical and periodontal tissues (7). Anderegg et al. reported 10 cases of palatogingival groove with significant periodontal destruction treated with guided tissue regeneration (GTR) using bio-bone materials and membranes, showing superior long-term outcomes (8). Tan et al. suggested intentional replantation for complex Type II or higher palatogingival groove cases, where the invaginated groove is reshaped and sealed with bio-materials *ex vivo*, isolating the groove from the pulp and periodontal tissues, achieving good coronal sealing, effectively controlling infection, and preserving the affected tooth (9). In summary, the treatment of palatogingival groove requires a comprehensive consideration of the lesion's severity, patient's age, and physical condition to develop a personalized treatment plan (10).

The treatment of Type III palatogingival groove is particularly challenging, requiring precise surgery to fill deep grooves and restore normal tooth morphology while considering post-treatment stability and aesthetics. This case report details the use of root canal treatment combined with intentional replantation to treat a maxillary lateral incisor with Type III palatogingival groove, with a 3-year follow-up to verify the efficacy and mid-term stability of this method, providing reference for similar cases.

## 2 Case report

### 2.1 Preoperative examination

A 35-year-old male patient presented with a chief complaint of “pus discharge from the upper left anterior tooth for 3 days.” The patient reported a history of persistent throbbing pain and biting discomfort in the affected tooth lasting 1 week, approximately 1 year prior. This was followed by recurrent gingival swelling with purulent discharge. Symptom relief was achieved with oral administration of metronidazole. He had no known drug allergies, and no family history of similar dental issues. The patient maintained good oral hygiene by brushing twice daily, although the use of dental floss was infrequent.

**Figure 1:**
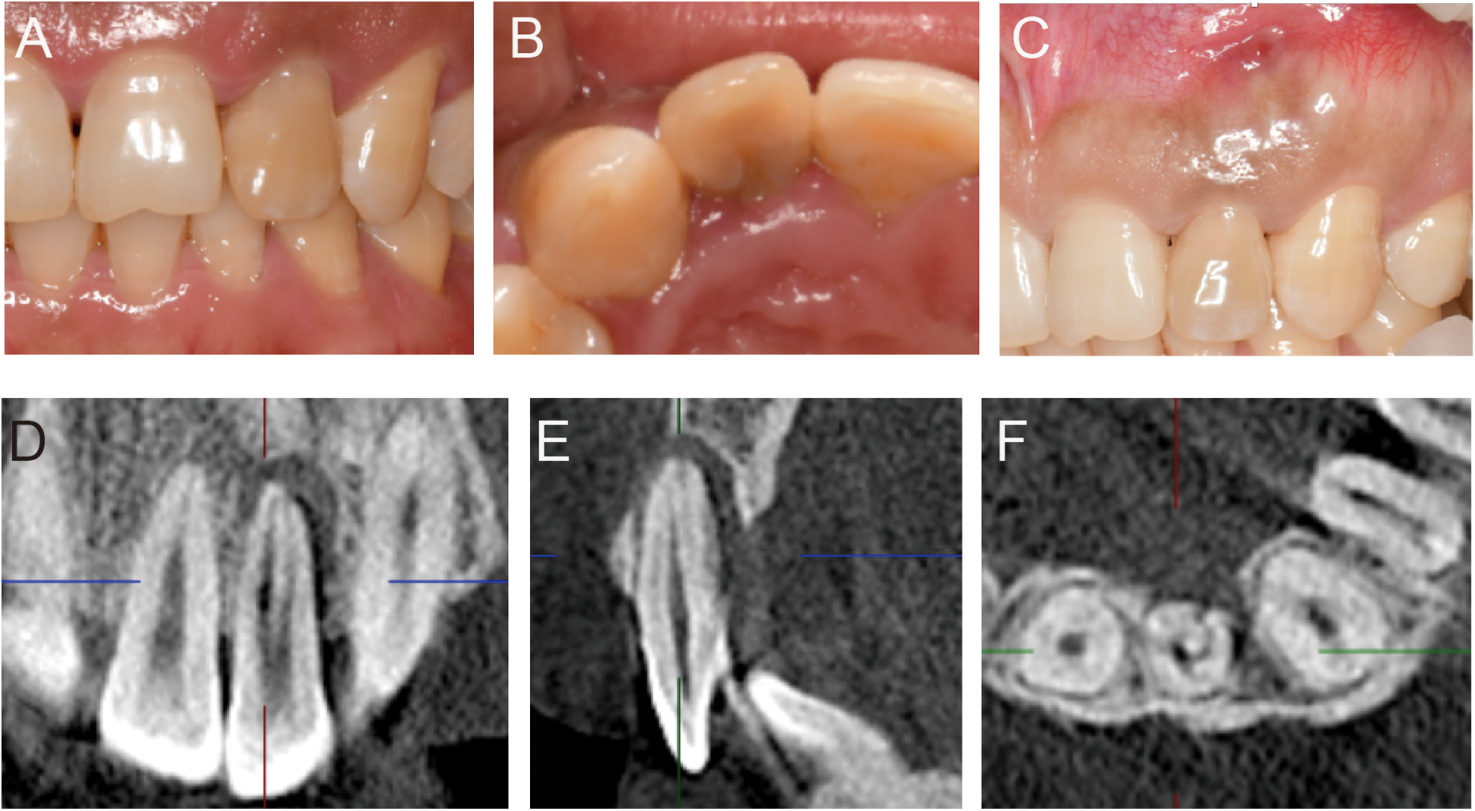
Preoperative clinical intraoral photos and imaging results. **(A)** Labial view of tooth 22 showing an intact but discolored crown. **(B)** Palatal view of tooth 22 revealing a malformed lingual groove extending from the lingual fossa to below the gingiva. **(C)** Labial view of tooth 22 showing a residual sinus tract at the apical region. **(D–F)** CBCT results showing the malformed lingual groove on the palatal side extending to the apical area and connecting with a low-density image in the periapical region. The root canal is empty and exhibits a C-shaped configuration.

The upper left lateral incisor (tooth 22) exhibited a discoloured crown without obvious caries. The mucosa at the labial apex showed a residual fistula with tenderness on palpation. A subgingival groove extending from the lingual tubercle to the apex was evident on the palatal side, with a probing depth of 10 mm and bleeding on probing. Probing depths on the mesio buccal, buccal, disto buccal, disto palatal, and mesio palatal sides ranged from 2 to 3 mm. The tooth was sensitive to percussion and palpation but had normal mobility within physiological limits. Cold stimulus testing showed no response, and electric pulp testing indicated a value of 80 compared to 8 for the control tooth (Figure 1A–C).

Cone beam computed tomography (CBCT) was used to further assess the tooth and surrounding bone structures. The CBCT revealed complete root development of the upper left lateral incisor, with a missing labial cortical plate at the apex, a thin residual cortical plate, localized palatal alveolar bone resorption, and a connecting lesion between the palatal and apical areas. The coronal view showed double root canals, and the horizontal view revealed a radiolucent C-shaped line extending from the pulp chamber to the periodontal ligament space (Figure 1D–F).

The diagnosis was Type III palatogingival groove with a combined periodontal-endodontic lesion in tooth 22. The first treatment option was root canal treatment followed by intentional replantation to preserve the tooth. The second option was tooth extraction followed by orthodontic or implant restoration. After a detailed explanation of the treatment process, potential risks, and expected outcomes, the patient opted for the first option and signed an informed consent form.

### 2.2 Surgical preparation and procedure

**Figure 2:**
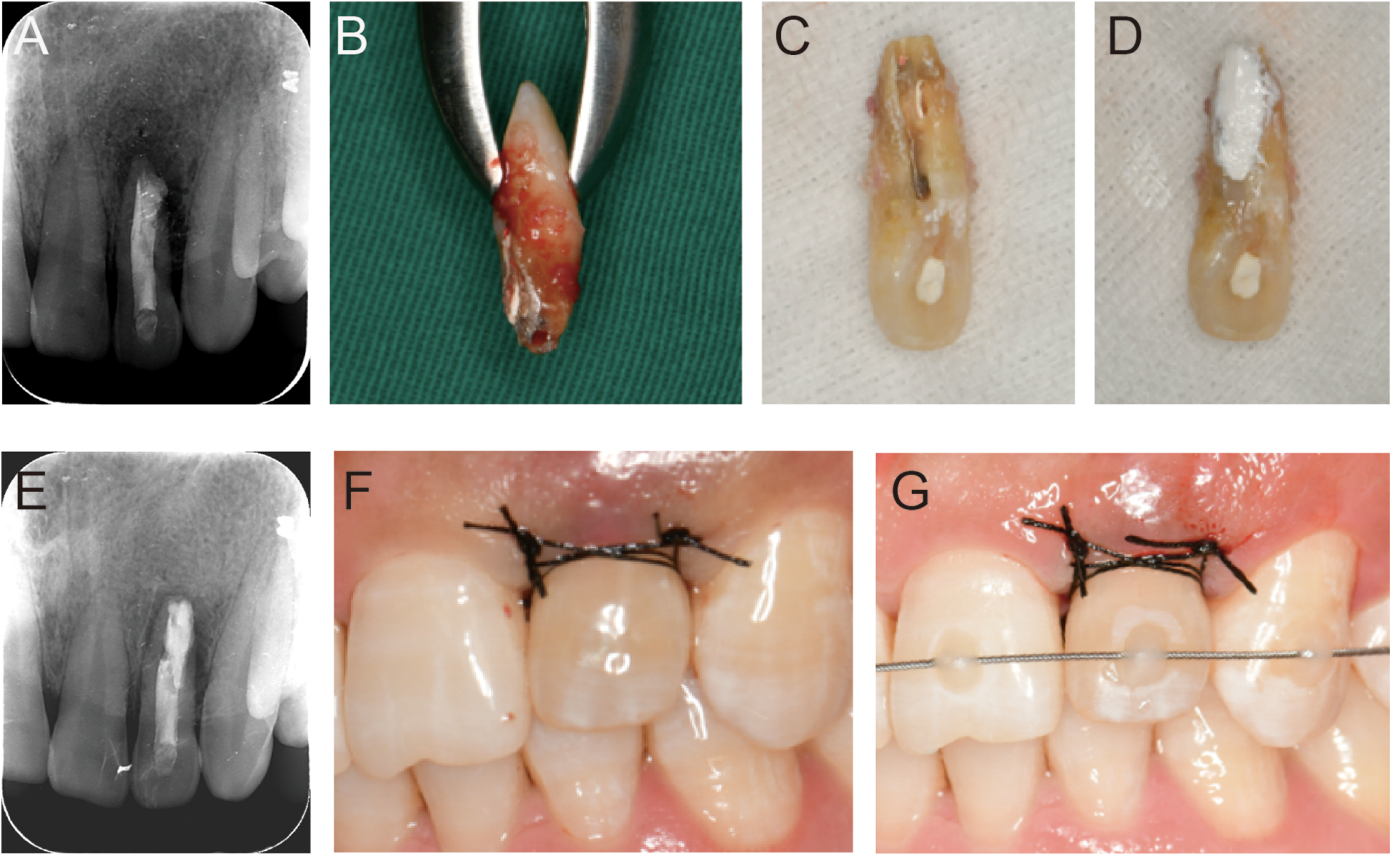
Intraoperative clinical intraoral photos and imaging results. **(A)** Immediate postoperative radiographic examination after root canal filling shows slight overfilling of the sealer. **(B)** Palatal view of tooth 22 after extraction, revealing the malformed central groove extending to the apical area, with overfilled sealer and black soft caries visible on the central root surface. **(C)** After preparation of the malformed central groove and root surface, the malformed groove and perforation area on the root surface are clearly visible. **(D)** The malformed groove is filled using a segmented approach. **(E)** Immediate postoperative radiographic examination after replantation. **(F,G)** Suturing and labial elastic fixation of tooth 22 following replantation.

Root canal treatment was initiated on the affected tooth under rubber dam isolation. The crown-down technique was employed for canal preparation using ProTaper system (Dentsply Maillefer, Ballaigues, Switzerland), shaping the canals up to F3 file (No.30/0.06), followed by further apical preparation to size #60 using hand K-files (Dentsply Maillefer). Copious irrigation with 2.5% sodium hypochlorite solution was performed, and calcium hydroxide was placed as an intracanal medicament during the inter-appointment interval. One week later, the tooth was re-accessed for cleaning, and final irrigation was performed with 17% ethylenediaminetetraacetic acid (EDTA). The root canals were obturated using the warm vertical compaction technique with injectable root canal sealer (iROOT SP, Henry Schein), and the coronal cavity was sealed with a zinc oxide base and temporarily restored with glass ionomer cement (Figure 2A). The patient was advised to maintain good oral hygiene, and intentional replantation was scheduled 1 week later.

Local infiltration anesthesia with 4% Articaine and 1/100,000 epinephrine was administered. The affected tooth was carefully extracted using a minimally invasive extraction forceps, and the integrity of both the root and crown was thoroughly examined under the microscope. The central palatal groove extended from the lingual tubercle to the apical foramen, revealing black soft caries and residual root canal sealer (Figure 2B). The groove and root surface were cleaned using a turbo diamond bur and ultrasound, and the apical 3 mm was removed (Figure 2C).

To seal the malformed lingual groove, a segmented filling approach was used. Beautifil Flow Plus F00 (Shofu Dental Corporation, Osaka, Japan) sealed the defective coronal part at the enamel junction. The root part, including the root surface invagination, root surface defect, and apical retrofilling, was filled with a nano-bioactive material (iRoot BP Plus, Innovative Bioceramix Inc, Vancouver, Canada) to prevent reinfection (Figure 2D). Throughout the procedure, the tooth was kept moist with saline-soaked gauze. The total extraoral operation time was approximately 5–6 min. The tooth was then replanted into the extraction socket and stabilized with an orthodontic stabilizing archwire (CO-AX 0.18) for labial elastic fixation (Figure 2E–G).

### 2.3 Postoperative follow-up

**Figure 3:**
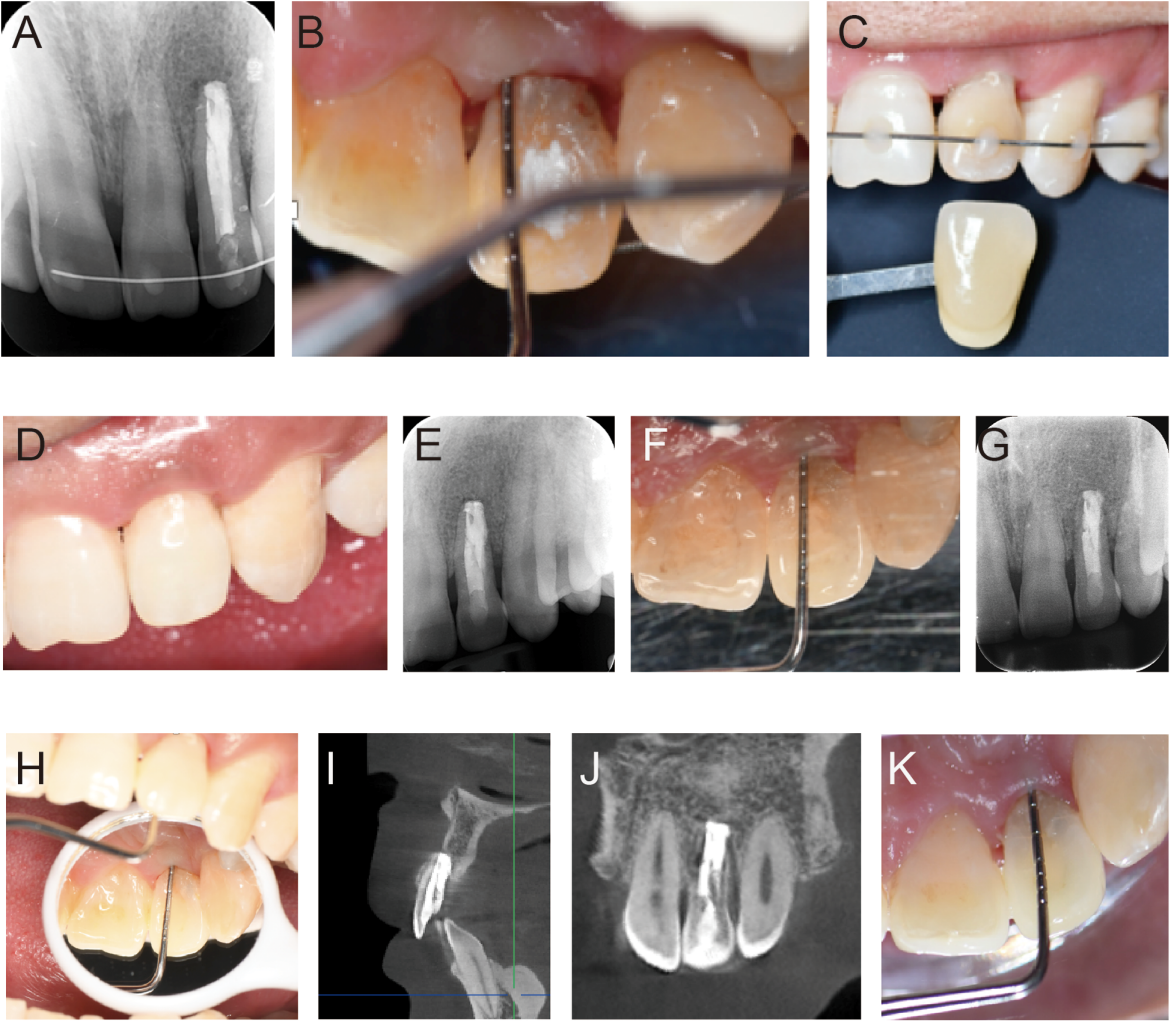
Postoperative intraoral photos and imaging results from regular follow-ups. **(A)** Radiographic examination results at 2 months postoperatively. **(B)** At 2 months postoperatively, the probing depth in the central palatal area of tooth 22 was reduced to 2 mm. **(C,D)** Tooth discoloration of tooth 22, shade matching, and post-internal bleaching show the crown color is consistent with adjacent teeth. **(E,F)** Radiographic examination and palatal probing depth check at 12 months postoperatively. **(G,H)** Radiographic examination and palatal probing depth check at 24 months postoperatively. **(I–K)** Radiographic examination and palatal probing depth check at 36 months postoperatively.

On the day of surgery, the patient reported only mild postoperative pain, with no discomfort from the second day onwards. Two months after replantation, the sinus tract had healed, the periodontal tissue's color and texture returned to a healthy state, the palatal probing depth reduced by approximately 8 mm without bleeding or pus discharge, and the tooth showed no mobility. Radiographic examination showed the disappearance of low-density radiolucency at the apex and new bone formation (Figure 3A,B). The labial stabilizing archwire was removed at this time.

Tooth #22 was recorded as shade 3M2 on the VITA shade guide, showing visible discoloration compared to the adjacent tooth #21, which matched shade 2M2 (Figure 3C). Internal bleaching was then performed using Opalescence Endo (Ultradent Products Inc., South Jordan, USA). Post-treatment, the color of the crown was harmonized with that of the adjacent teeth (Figure 3D). Follow-ups at 12, 24, and 36 months showed no symptoms, no mobility, and a palatal probing depth of 2–3 mm. CBCT at the 3-year follow-up revealed dense root filling material with good sealing, gradual recovery of the periodontal membrane at the apex, and formation of new periodontal attachment without signs of ankylosis or external inflammatory root resorption (Figure 3E–K).

## 3 Discussion

Palatogingival groove, a complex dental anomaly, presents significant challenges in dentistry. It may result from genetic factors, developmental abnormalities, or early trauma (11). Type III palatogingival groove is particularly challenging, leading to periodontal tissue loss, deep periodontal pockets, and an ideal environment for bacterial growth, which can result in pulp necrosis, periapical infection, and periodontal disease (12, 13). Early diagnosis is essential, with CBCT playing a critical role in identifying the groove and its relationship with surrounding tissues, enabling precise treatment planning (14, 15). When combined with microscopy and ultrasonic technology, CBCT acts as a digital navigation tool, minimizing surgical risks and improving treatment outcomes (16). Although case reports on palatogingival groove have increased in recent years, medium- to long-term follow-up studies are lacking. This study provides mid-term follow-up on a Type III palatogingival groove case, providing insights into its pathogenesis, treatment challenges, and management strategies.

This case describes a type III maxillary lateral incisor with a palatogingival groove, presenting combined periodontal and endodontic lesions. Preoperative CBCT revealed narrow bone width, an unfavorable location of the labial sinus tract, severe palatal bone resorption, and uncertain long-term implant stability. Given the patient's desire to retain the tooth, root canal treatment combined with intentional replantation was performed. The dual infection source in the root canal and invaginated groove had rendered single-method treatments ineffective, often leading to extraction within 5–6 months (17). Some scholars have attempted to preserve such teeth using microsurgical apicoectomy combined with guided tissue regeneration (GTR), but in cases like this, where the root surface groove extends to the apex and severe bone defects are present, the increased surgical difficulty and trauma from extensive bone removal pose significant risks (3). Intentional replantation has shown favorable clinical outcomes, standing out compared to periodontal flap surgery, GTR, or apical surgery (9). It allows thorough debridement and repair under direct vision, effectively eliminating the infection source and promoting tooth healing. The technique minimizes contamination risks by controlling the extraoral operation time within 15 min and maintaining root moisture with Hank's balanced salt solution, significantly reducing root resorption. Long-term success rates (>93%) and healing outcomes (72%–91%) are comparable to non-surgical retreatment and apicoectomy (18, 19).

Effective control of periodontal and periapical infection is critical for successful tooth replantation. In this case, preoperative use of chlorhexidine mouthwash reduced the bacterial load, while thorough root canal disinfection eliminated potential infection sources. The choice of root canal filling material is crucial for restoring tooth structure and controlling infection. iRoot BP Plus was selected to seal the root surface groove due to its excellent marginal sealing, stability, and biocompatibility. Upon contact with body fluids, it forms a hydroxyapatite-like layer, creating a favorable environment for osteoblast and periodontal ligament growth (20). Flowable resin was applied to seal defects above the enamel-dentin junction, ensuring effective sealing and minimizing plaque accumulation (21). The C-shaped root canal was thoroughly cleaned using microscopy and ultrasonic irrigation to preserve tooth structure and remove intracanal infections (22). Although a secondary canal near the malformed groove may have been overlooked, the external sealing remained intact. Regular follow-up is necessary, and if the external sealing is compromised in the future, intervention through root canal retreatment or flap surgery may be required.

Periodontal tissue recovery is a key indicator of treatment success, as it relates to the tooth's function and stability. In this case, the patient experienced mild pain on the second day post-surgery, with no subsequent discomfort during follow-up, indicating a favorable initial response. Over 3 years, periodontal probing depth and tooth mobility remained stable, with no signs of ankylosis or pathological root resorption, demonstrating good periodontal health. At the 1-year follow-up, minor gingival papilla recession (∼1 mm) and mild black triangle formation were observed (Figure 3D), likely due to alveolar bone remodeling after replantation (23). These issues remained stable at the 2- and 3-year follow-ups, and the patient reported no aesthetic concerns. For further improvement, membrane graft surgery or veneer restoration could be considered if necessary. Discoloration of the affected tooth, caused by pulp necrosis and blood decomposition, was another concern (24). The patient expressed a desire to improve the aesthetics of the affected tooth at the initial visit. Internal bleaching with 35% hydrogen peroxide successfully improved tooth color and restored smile aesthetics, aligning with studies showing comparable outcomes between 35% hydrogen peroxide and carbamide peroxide after 14 days (25). However, the risk of discoloration recurrence due to marginal leakage or permeability remains, necessitating regular follow-up to maintain optimal outcomes (26).

## 4 Conclusion

This case report presents a complex case of palatogingival groove. Through mid-term follow-up, we have gained a deeper understanding of the pathogenesis, treatment challenges, and effective methods for managing Type III palatogingival groove. The extended follow-up confirmed the stability and reliability of this treatment approach. We believe that in future practice, we will be able to offer more comprehensive, precise, and personalized treatment plans for patients with palatogingival groove, helping them overcome the condition and restore a healthy oral state.

## Data Availability

The original contributions presented in the study are included in the article/Supplementary Material, further inquiries can be directed to the corresponding author.
